# Mitigating mutual coupling effects on circular polarization for improved bandwidth in MIMO systems: A novel approach

**DOI:** 10.1016/j.heliyon.2024.e27782

**Published:** 2024-03-12

**Authors:** A. Ali, M.N.M. Yasin, I. Adam, A.M. Ismail, S.P. Jack, Abdullah Alghaihab, N.I.M. Nor, N.A.A. Rahman

**Affiliations:** a Advance Communication Engineering (ACE), Centre of Excellence, Universiti Malaysia Perlis (UniMAP), Kangar, Perlis, 01000, Malaysia; b Faculty of Electronic Engineering & Technology, Universiti Malaysia Perlis, 02600, Arau, Perlis, Malaysia; c Faculty of Information Technology and Electrical Engineering, University of Oulu, P.O. Box 4500FI-90014, Oulu, Finland; d Department of Electrical Engineering, College of Engineering, King Saud University, P.O. Box 800, Riyadh, Saudi Arabia

**Keywords:** Circularly polarized (CP), Characteristic mode analysis (CMA), Dielectric resonator antenna (DRA), Mutual coupling (MC)

## Abstract

An improved mutual coupling compensation in circularly polarized (CP) multi-input multi-output (MIMO) dielectric resonator antenna (DRA) is presented in this paper. Using trimming approach, the mutual coupling (MC) between closely spaced DRA units at 0.3λ has been significantly reduced while axial ratio performance has been maintained. Mutual coupling reduction is obtained by trimming the DRA to ensure low mutual coupling below −20dB. The exclusive features of the proposed MIMO DRA include wide impedance matching bandwidth (BW), triple band circular polarization, and suppressed MC between the radiating elements. The impedance bandwidth matches perfectly with a triple band's 3 dB axial ratio (AR). It is designed with characteristic mode analysis with good agreement of the measurement that has been obtained. Using the probe feed method, the DRA and patch strip are coupled together to allow bandwidth widening of the pro-posed DRA. An impedance bandwidth of 34% at a lower frequency to around 2% at a higher frequency was achieved in all resonance frequencies. Thus, we refer to our newly designed DRA as a proposed method for effectively reducing the mutual coupling between DRAs. Additionally, the 3 dB AR bandwidth matched at 3.3 GHz, 4.6 GHz, and 6.3 GHz with a percentage of 11.66%, 3.04%, and 2.22% obtained at the three different frequencies. Note that the proposed DRA exhibits low mutual coupling (below −20 dB) at the targeted frequencies, which is suitable for better signal reception for MIMO applications. By computing, the metrics envelop correlation coefficient, diversity gain, channel capacity loss, and total active reflection coefficient, the MIMO performance of the proposed antenna is verified. The experiments show a close result between simulated and computed validation of the proposed DRA.

## 1 Introduction

Circularly polarized (CP) band antennas are crucial in modern wireless systems for their ability mitigate the impact of the multipath effect and its associated losses from air reflection and wave absorption. Moreover, the utilization of CP waves has been shown to result in improved signal transmission properties in comparison to linearly polarized (LP) waves. Despite the challenges posed in generating CP waves, the demand for their production has grown with the rapid expansion of wireless communication services, which require higher transmission rates and larger transmission capacities. However, the current hindrance to the advancement of wireless communication lies in the limited frequency band availability. To make the most of the finite frequency spectrum, the development and extensive exploration of multiple-input-multiple-output (MIMO) technology has been undertaken. MIMO technology offers the potential to significantly enhance channel capacity while conserving spectrum resources [[1], [2], [3]].

In practical communication systems, the available space for a MIMO antenna is often limited. The mutual coupling between the antenna components of a MIMO system can significantly degrade its performance. To address this issue, researchers have been seeking methods to mitigate mutual coupling between the antenna components. In this regard, the integration of CP and MIMO antennas have been proposed as a solution, due to the combined benefits of both technologies in terms of high transmission rate and reliable communication. The integration of CP and MIMO antennas has received growing attention from researchers, though the number of active researchers in this field remains limited [[4], [5], [6], [7], [8]].

An investigation [4] introduced the implementation of an electromagnetic bandgap (EBG) structure to address mutual coupling issues between pairs of CP du-al-ridged antennas. A mu-negative metamaterial filter-based decoupling method was proposed for CP MIMO monopole antennas in Ref. [5], while a two-layer transmission-type frequency selective surface was utilized in Ref. [6] to achieve decoupling of CP patch antennas. The deployment of decoupling structures between or above the antenna elements in all three of these designs leads to an increase in system complexity and height. In Ref. [9] the combination of parasitic patch and diagonal positioning of radiating elements to improve bandwidth and suppress mutual coupling is suggested. However, this approach requires more space and limited distance, making it unsuitable for practical systems. Additionally, the diagonal positioning of dual-ridged antennas results in a larger system hardware size, which is not ideal for MIMO applications. To date, some recent works using multi-mode approach in cavity structure [10] to enhance the CP bandwidth but still the design are complicated and bulky.

For this paper, a new and practical method to improve isolation between identical CP DRAs is proposed, featuring a triple-band CP antenna fed by a coaxial cable. The proposed method presents a novel and effective solution for obtaining a triple-band CP antenna with excellent axial ratio bandwidth. Without the need for external materials or intricate DRA design, the parasitic and external stub effects offer a distinctive technique for realizing a broad impedance bandwidth in a higher mode [2]. By strategically trimming the DRA structure, the proposed approach realizes triple-band CP and enhances the antenna's gain performance.

The isolation between two CP dual-ridged antennas (DRAs) was significantly improved by 20–30 dB at resonance frequencies. The performance of the CP DRAs, including reflection coefficient, radiation pattern, and axial ratio (AR), remained stable and uncompromised when operating individually or simultaneously. The structure of this article is outlined as follows: In Section II, the configuration and decoupling mechanism of the proposed CP MIMO DRA are described. Section III presents measurement verification. The article concludes in Section IV. The biggest challenge in de-signing a triple-band CP lies in the need to attain both high AR and impedance matching for all three bands. To achieve optimal performance for MIMO applications, mutual coupling must also be reduced to its lowest possible value.

The simulation of antenna characteristics, including radiation patterns, input impedance, and radiation efficiency, was performed using CST Microwave Studio. The theory of Characteristic Mode Analysis (CMA) is employed to forecast the performance and behavior of antennas [8]. This approach provides valuable insight into modal performance, enabling predictions of bandwidth and radiation potential, as well as eigen currents for forecasting coupling modes and characteristic angles for predicting antenna resonance mechanisms. The CMA can determine the basic modes' resonant frequencies and higher-order modes' resonant frequencies in the absence of activation. Additionally, it specifies each mode's electromagnetic resonance characteristics [11].

The simulated and measured characteristics of the return losses, AR, radiation patterns, and gain have been analyzed. The simulated results match closely with the measured ones, confirming the originality of the proposed MIMO DRA design. No research has been found that uses this design without additional material or complexity and still achieves triple-band CP and improved isolation.

## 2 Materials and methods

**Fig. 1:**
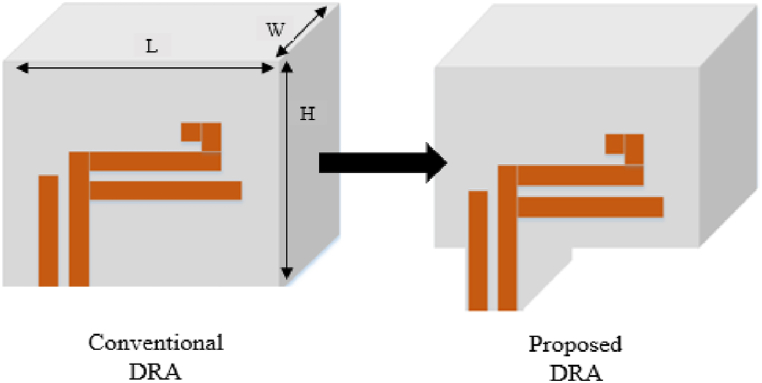
Single element of proposed design.

Before designing the MIMO DRA, a single DRA was designed and its performance validated. The RDRA was made of alumina (ECCOSTOCK) with a relative permittivity of 10 and dimensions of H = 26.1 mm, L = 21.3 mm, and W = 14.3 mm. The triple-band CP was achieved through optimization of the DRA structure by trimming, as shown in Fig. 1. A parasitic patch was added to improve the return loss and CP wave band-width.

The details of the CMA theory [12] have been omitted for brevity. Only two key quantities - the characteristic angle, α and the modal significance (MS) as a function of eigenvalue, λ are defined in (1) and (2), respectively, as follows [6,7]:

(1) $$\alpha =180^{o}\;–\tan^{-1}\left(\lambda \right)$$

(2) MS=1/(|1+jλ|)

MS is the normalized amplitude of the characteristic current, with a value greater than 1/√2 indicating a significant mode. The Characteristic Angle (CA) is the phase difference between the actual characteristic current and its associated characteristic field, providing insight into the behaviour of each CM. A 90° CA difference between two orthogonal modes is required for CP radiation, which can be achieved by exciting the two modes simultaneously. The CA physically describes the phase difference between a characteristic current and its associated characteristic field. A mode is considered in resonance when its CA is close to 90°. The MS measures the contribution of a specific mode to the total radiation when a source or excitation is applied [[13], [14]].

**Fig. 2:**
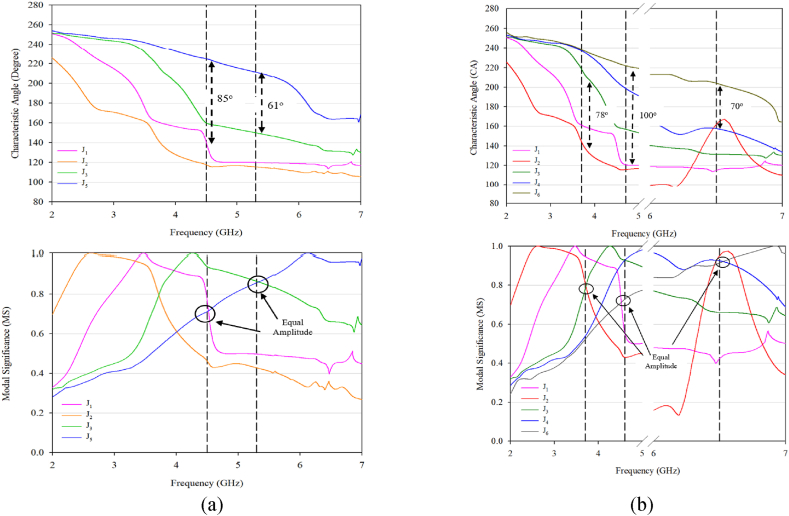
Characteristic angle and modal significance: (a) conventional (b) proposed design.

The CMA was performed on both designs over a frequency range of 2–7 GHz, using a maximum of 5 modes to describe the electromagnetic behaviour of the antenna. The other modes are considered higher-order and difficult to resonate. Fig. 2 displays the CA and MS of the 5 modes. The analysis found that for the conventional design, two modes have the same current amplitude and a 90° phase difference at two different frequencies (around 4.6 and 5.3 GHz). Meanwhile, the proposed design resonates at three different frequencies (around 3.3, 4.6, and 6.3 GHz) with the same current amplitude and phase difference.

The conventional design has four dominant modes that generate CP radiation, which are Modes 1 and 4 (J1 and J4) at 4.6 GHz with a CA around 85°, and Modes 3 and 5 (J3 and J5) at 5.3 GHz with a CA around 61°. This information can be seen in Fig. 2(a), which displays the CA and MS for the conventional design. On the other hand, for the pro-posed design (Fig. 2(b)), different modes are activated at different frequencies.

For example, at 3.5 GHz, Modes 1 and 2 (J1 and J2) can be easily excited with a 78° CA difference, making them capable of generating CP radiation. At 4.6 GHz, Mode 1 and 6 (J(b1 and J6) are the dominant modes, resulting in the potential to generate circular polarization (CP) radiation, due to the 100° CA difference between them. At a higher frequency, four modes are activated with equal amplitudes as shown in MS, but Mode 4 and Mode 6 (J1 and J3) with 70° CA difference are the active modes, still leading to CP radiation.

**Fig. 3:**
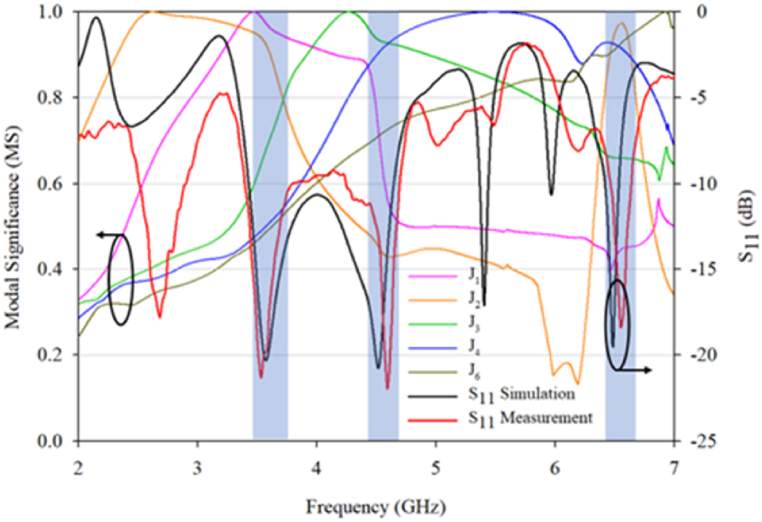
Modal significances and reflection coefficients of the excited proposed design characteristic angle and modal significance.

The measurement results as in Fig. 3 confirm the predictions made by the CMA. The three resonant frequencies are in close agreement with the simulation results with errors less than 1% and Fig. 3 show the MS and reflection coefficients of the excited frequency match with each other. This indicates that the proposed design has a strong potential to generate circular polarization radiation at respective frequencies.

Table 1 compares the 3 dB AR bandwidths of the two designs, showing that the proposed design has wider bandwidths and generates another CP at a higher frequency centered at around 6.49 GHz. This means that the proposed design has better performance compared to the conventional design.

**Table 1:** Comparative results of AR for conventional and proposed DRA.

No	Conventional DRA	Proposed DRA
1	10.27% (3.95–4.38 GHz)	11.27% (3.3–3.68 GHz)
2	0.53% (5.28–5.31 GHz)	12.18% (4.17–4.69 GHz)
3	–	1.74% (6.44–6.55 GHz)

**Fig. 4:**
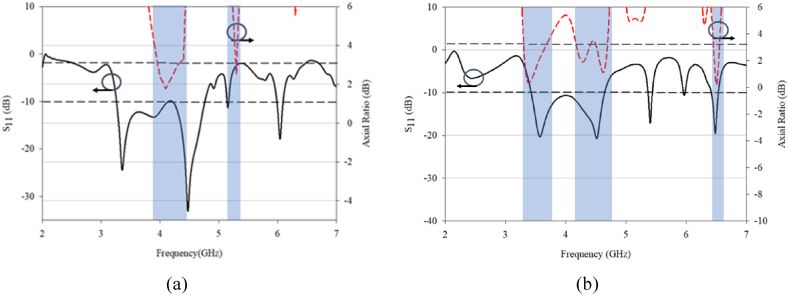
S11 and 3-dB axial ratio (AR) characteristics for: (a) Conventional; (b) Proposed DRA.

Fig. 4 presents the overlapping impedance BW with 3 dB AR BW. Apparently, the proposed DRA demonstrated superior performance over the conventional DRA, mainly because the overlapping impedance matched at three different frequencies. This indicates that the proposed design can provide better impedance matching. It means that the proposed design can transfer more energy from the source to the antenna and minimize the reflection loss. This result indicates that the proposed design can achieve better radiation performance than the conventional design.

In conclusion, the proposed design of DRA demonstrated superior performance compared to the conventional DRA. This is because the proposed design combined parasitic patches and trimmed a specific area, which improved the performance without the need for added structure. The results of the study were obtained through CMA and overlapping impedance bandwidth with 3 dB AR bandwidth investigation. The proposed design was then selected for further analysis in MIMO systems.

## 3 MIMO antenna analysis

**Fig. 5:**
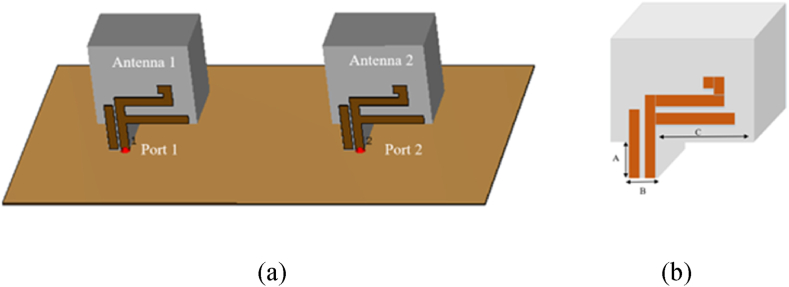
Structural view of the proposed MIMO DRA: (a) Perspective view; (b) Close view of DRA.

The dual-port dielectric resonator MIMO antenna design involves the use of a single DRA placed in the middle of a ground plane made of PEC (Perfect Electric Conductor) material. The DRA is excited using the probe feed technique, and the design of the parasitic patch shape is the same as in the single element section. This configuration aims to provide two separate ports for MIMO communication. The layout of the proposed MIMO antenna can be seen in Fig. 5. The dimensions of the trimmed dielectric resonator used in the design are listed in Table 2. The design and dimensions of the DRA are crucial for optimizing the antenna performance.

**Table 2:** Dimension of trimmed DRA.

Label	Dimension (mm)
A	4.68
B	1.83
C	6.63

**Fig. 6:**
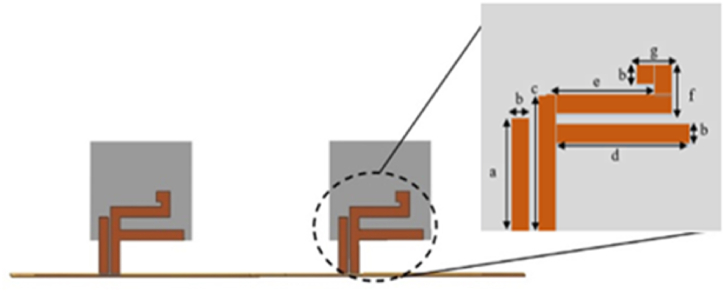
Front view with parasitic patch dimension.

Fig. 6 provides the details of the parasitic patch used in the MIMO antenna design. The dimensions of the parasitic patch are given in Table 3, which play an important role in determining the antenna's performance characteristics. These dimensions should be carefully chosen to ensure that the antenna operates within the desired frequency range and meets the specified performance requirements.

**Table 3:** Dimension of parasitic patch.

Label	Dimension (mm)	Label	Dimension (mm)
a	11	e	11
b	2	f	5
c	13	g	3
d	14		

**Fig. 7:**
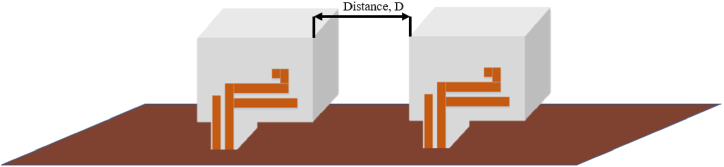
Distance variation for MIMO.

The design is simulated for MIMO application with separation of distance, *D*, as shown in Fig. 7. The distance is set from the edge of the first DRA to the edge of the second DRA. The distance variation is varied from 0.30λ (20 mm) to 0.50λ (40 mm) with an increment of 0.10λ (10 mm).

The study is divided into several subsections to comprehend the behaviour of the proposed MIMO antenna. The first comparison is made on conventional DRA with the proposed design. The parameters to focus on are the impedance bandwidth (BW), axial ratio (AR), and gain. The overlapping 3 dB AR bandwidth is the most important characteristic to consider as the performance of the DRA depends on the matching of both parameters as well as the generation of the circularly polarized wave.

### 3.1 Parametric study from conventional to the proposed structure

**Fig. 8:**
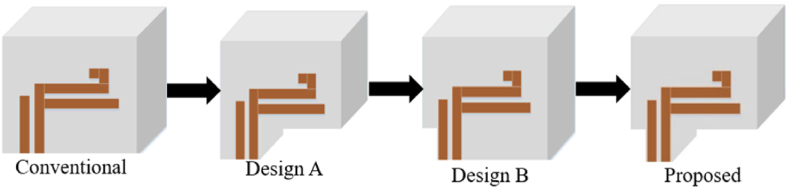
The improvement stage of the proposed DRA.

**Fig. 9:**
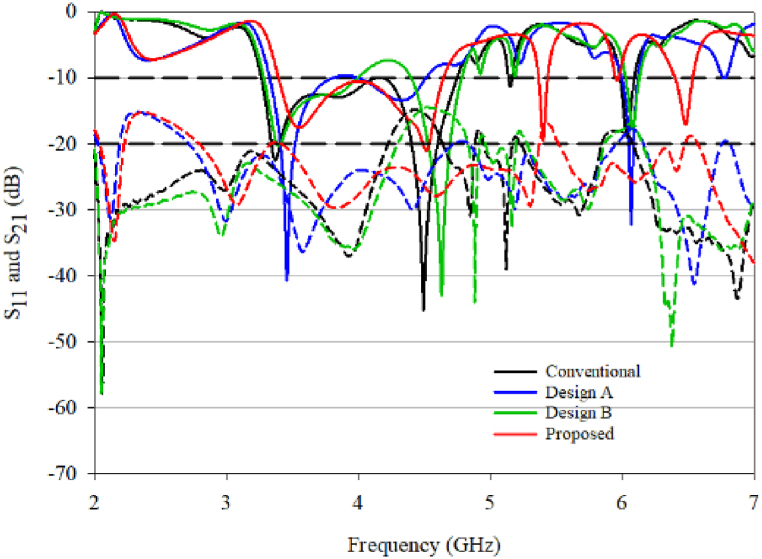
Comparison of simulated S_11_ and S_21_ for conventional, A, B, and proposed design.

The RDRA was then trimmed, as shown in Fig. 8, to reach the proposed design. The impedance bandwidth and 3 dB AR bandwidth were compared by examining the S-parameters (S_11_ and S_21_) and the AR as in Fig. 9. Both parameters must coincide at the same frequency for optimal MIMO system performance. The parasitic patch was left unmodified as adding it would not impact the MIMO performance. The parametric analysis of the patch was already completed in Ref. [15], and this paper uses an optimized design.

Trimming the DRA from the conventional design to designs A and B did not cause significant changes in S_11_ for low frequencies (3–5 GHz). However, design B showed a slight improvement in S_11_, with values less than −10dB in the lower frequency range of 4–4.4 GHz (represented by the green line). The value of S_11_ remained below −10 dB throughout. At higher frequencies, the proposed design experienced a slight shift in frequency to 5.4 GHz compared to the conventional, designs A and B. The proposed design demonstrated excellent S_11_ values (−17.22 dB) at 6.5 GHz, whereas the other designs failed to resonate at this frequency.

**Fig. 10:**
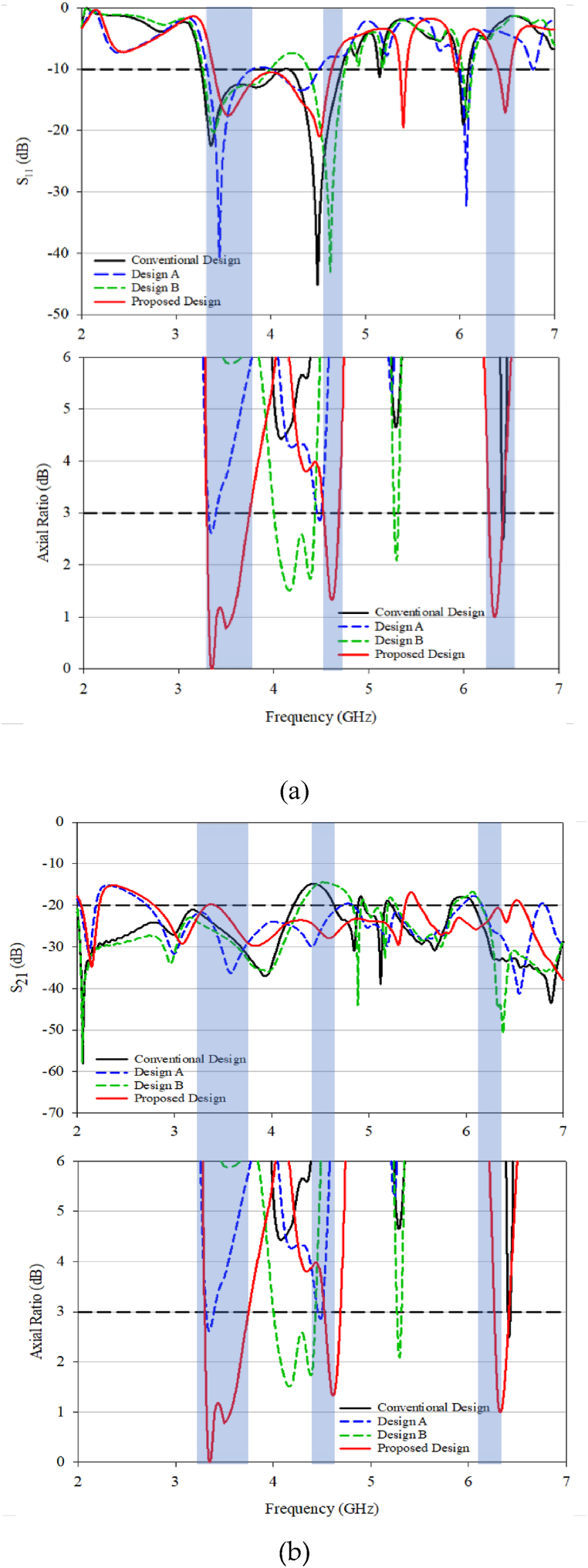
(a) S_11_ with axial ratio; (b) S_21_ with axial ratio.

A closer investigation comparing S_11_ and S_21_ with the axial ratio is presented in Fig. 10(a) and (b). Examining the S_21_ value, all designs exhibit favourable values below −20 dB. Trimming the DRA from the conventional design to designs A and B resulted in reduced mutual coupling, with design B approaching −40 dB, as shown in Fig. 10(b). The focus is on the resonant frequency, and the proposed design demonstrates better performance at lower frequencies (3–5 GHz) and higher frequencies (around 6–6.5 GHz) compared to the other designs.

The conventional design lacks overlap between the impedance BW and the 3 dB AR BW, whereas the proposed design displays three 3 dB AR BW. Designs A and B serve as reference designs for comparing the performance after trimming the DRA to obtain the proposed design. While both designs show a reduction in coupling, their impedance BW does not match with the 3 dB AR bandwidth.

### 3.2 Mutual coupling analysis for the conventional design

**Fig. 11:**
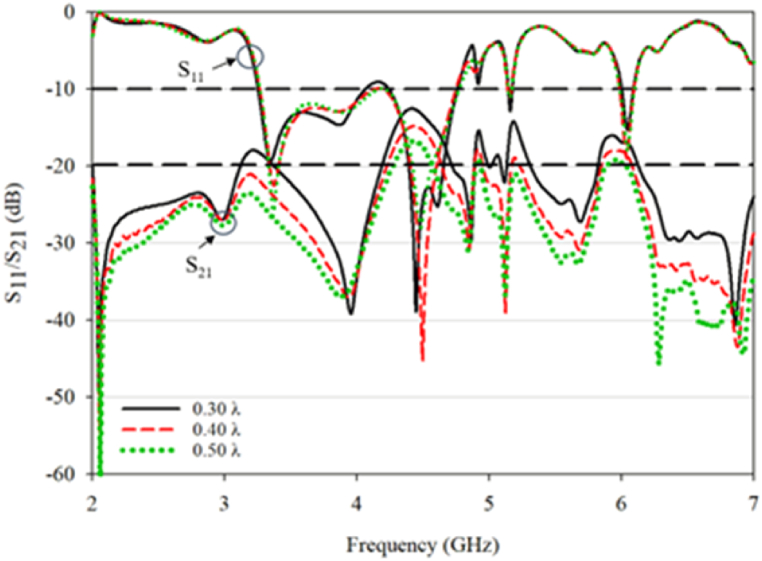
S_11_ and S_21_ for conventional DRA.

Fig. 11 displays the s-parameter values for the RDRA design with distance variations ranging from 0.30λ to 0.50λ. The conventional RDRA exhibits a return loss below −10 dB from 3.26 to 4.76 GHz, resulting in a triple-band impedance with 33.4% bandwidth centered at 2.08 GHz, 1.08% bandwidth centered at 5.16 GHz in the range of 5.12–5.18 GHz, and 1.67% bandwidth centered at 6.03 GHz from 5.99 to 6.09 GHz. Decreasing the distance between the DRA elements had no impact on the antenna's operating frequencies, but it resulted in increased coupling, which is undesirable in MIMO applications.

**Fig. 12:**
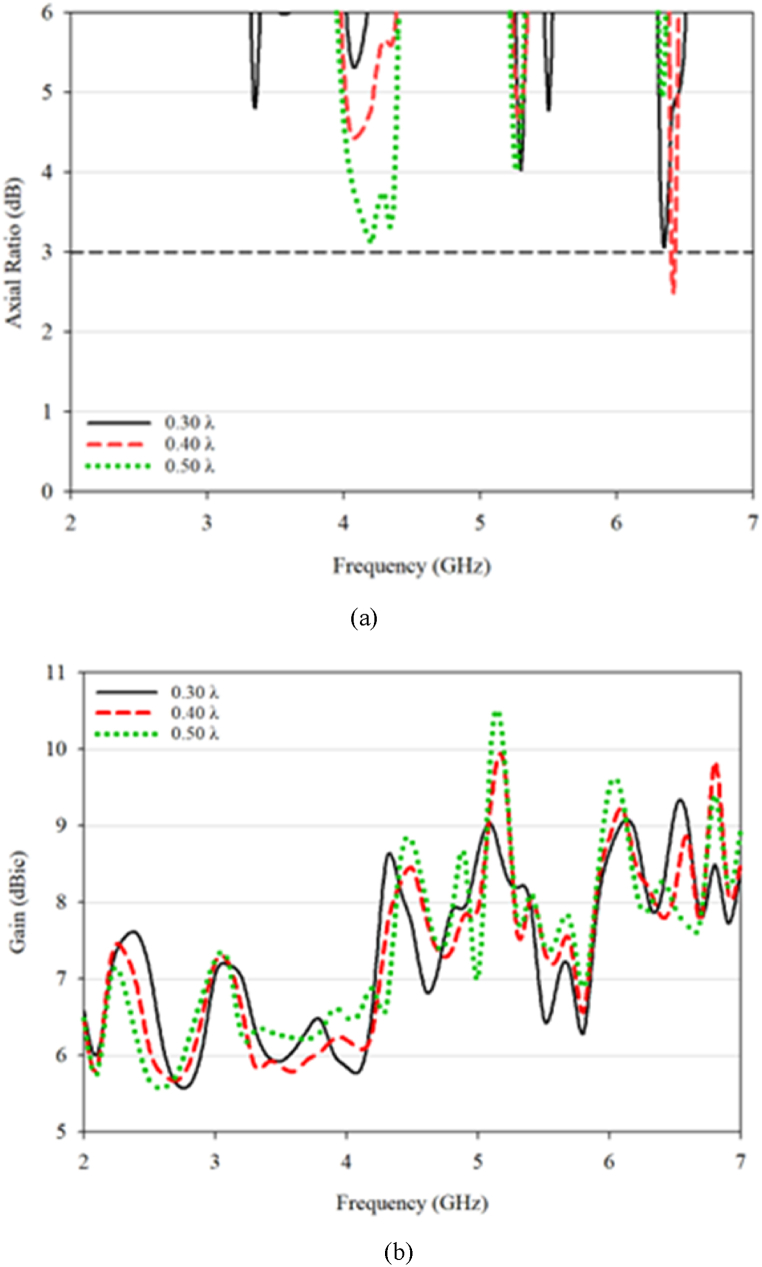
(a) Axial ratio; (b) gain value for conventional DRA.

Fig. 12 presents the simulated 3 dB AR bandwidth and gain for the RDRA. The conventional RDRA generates a single 3 dB AR bandwidth at a 0.40λ (30 mm) distance with a bandwidth of 2.01% (5.96–6.08 GHz) and 9.6 gain. The S_21_ value at the operating frequency is high, ranging from −10dB and above, and increases as the distance between the DRAs decreases. Thus, the conventional RDRA only exhibits one effective 3 dB AR, indicating that mutual coupling can potentially reduce CP formation at closer distances. Further optimization was performed on the proposed DRA structure to attain a triple-band CP with reduced mutual coupling.

### 3.3 Mutual coupling analysis for the proposed design

**Fig. 13:**
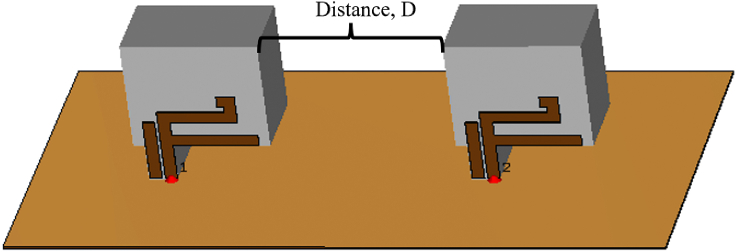
Perspective view of proposed DRA for MIMO.

An improvement was made from the conventional RDRA to the proposed structure by trimming the bottom of the DRA as depicted in Fig. 13. The bottom part of the DRA was trimmed, retaining only the feeder part of the parasitic element in contact with the ground plane. Simulation data was collected for the s-parameters, AR, and gain at distance variations from 0.30λ to 0.50λ.

**Fig. 14:**
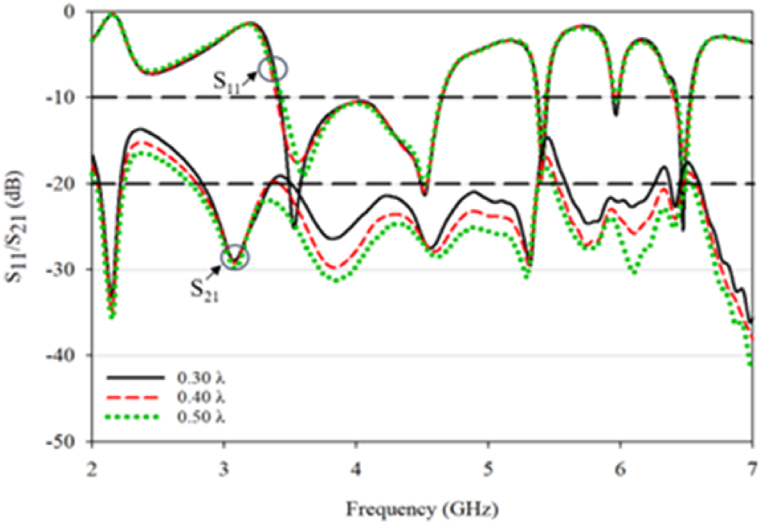
S_11_ and S_21_ for the proposed DRA.

The mutual coupling effect is the influence of one element of a system on another element in that same system. In this case, it refers to the impact of the proximity of the DRA on its performance parameters, such as AR and gain. The results in Fig. 14 show that the mutual coupling effect leads to a decrease in performance as the DRA gets closer in a confined space, with the lowest value being observed at a separation of 0.30λ. The proposed DRA has four frequency ranges with −10 dB impedance bandwidth. The first bandwidth is 34% and centered at 3.59 GHz, ranging from 3.41 to 4.65 GHz. The second bandwidth is 1.82% and centered at 5.4 GHz, ranging from 5.36 to 5.44 GHz. The third bandwidth is 0.8% and centered at 5.95 GHz, ranging from 5.93 to 6 GHz. The fourth bandwidth is 1.95% and centered at 6.47 GHz, ranging from 6.40 to 6.53 GHz.

**Fig. 15:**
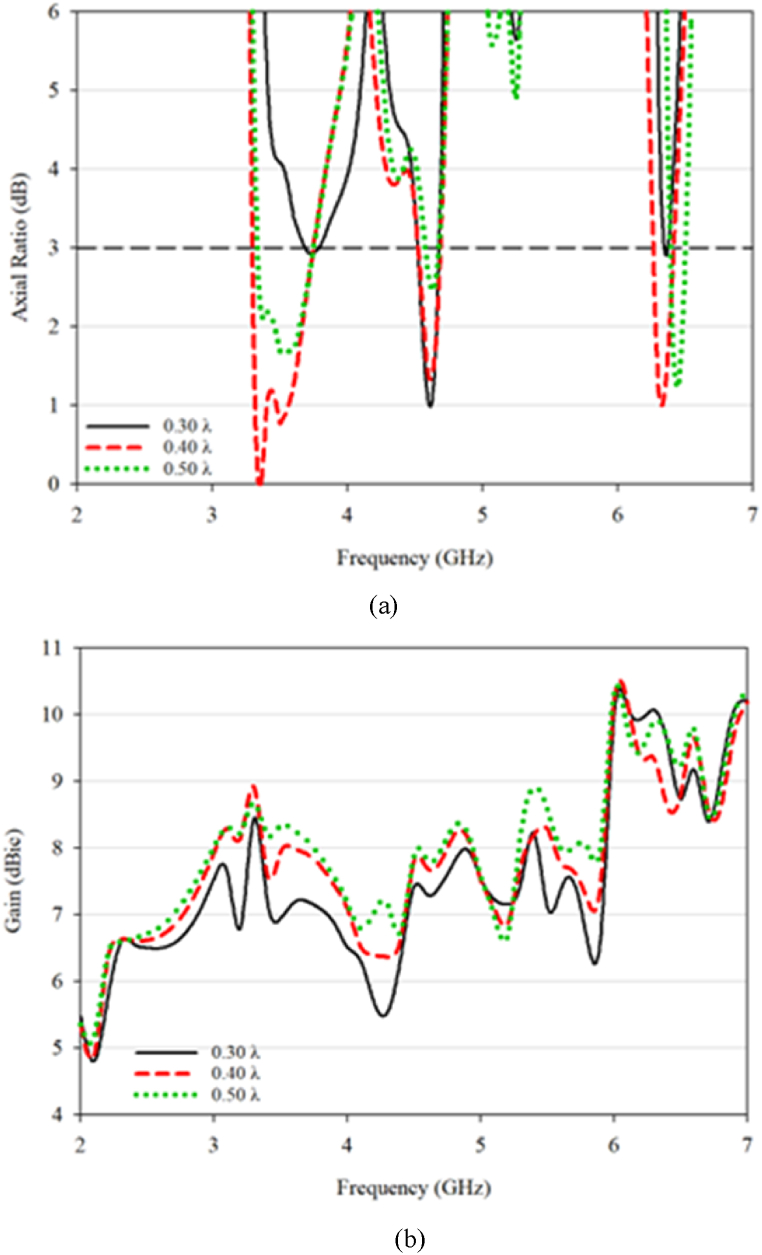
(a) Axial ratio; (b) gain value for the proposed antenna.

The proposed DRA has three 3 dB AR bandwidths, with the first one being 11.88% and centered at 3.54 GHz (between 3.33 and 3.75 GHz), the second one being 3.04% and centered at 4.62 GHz (between 4.55 and 4.69 GHz), and the third one being 2.22% and centered at 6.33 GHz (between 6.26 and 6.4 GHz). The gain of the proposed DRA is 8.3, 7.8, and 9.9 for the three 3 dB AR bandwidths, respectively. The value of S_21_ at the resonant frequency is below −20 dB. A comparison of the conventional and proposed DRA demonstrates that trimming the DRA can greatly reduce the mutual coupling impact and increase the overall performance of the MIMO system, as shown in Fig. 15.

In the previous study, single-element validation was conducted to by comparing the conventional and proposed design [15]. The conventional design is capable of generating only dual-band CP, while the proposed design can generate triple-band CP. The proposed design demonstrates enhanced capabilities in terms of CP generation, offering an additional CP band compared to the conventional design. This paper investigates the implementation of the two designs specifically for MIMO applications. The results showed that while the conventional design only produced one CP band, the proposed design maintained the triple CP band.

Furthermore, the investigation of the mutual coupling (MC) effect revealed that the proposed design effectively maintained the CP performance even with a significant reduction in MC compared to the conventional design. It is worth noting that, to the best of the author's knowledge, previous research has mainly focused on generating CP bands and maintaining CP in MIMO applications, with little attention given to the effect of MC on CP generation. Table 4 provides a summary of the MC reduction techniques employed in previous research studies that focus on a single CP band, in comparison to the present research that achieves triple-band CP generation. The table highlights the different techniques used in each study to mitigate the impact of mutual coupling on CP performance.

**Table 4:** Comparison from previous research for MC reduction MIMO.

Ref.	Decoupling structure	CP band	Isolation (dB)
26	DGS	1	−28
22	Eight vertical metallic strip	1	−31
27	Microstrip feed DRA	1	−22
28	Parasitic element	1	−38 to −48.5
This paper	Trim DRA	3	−20 to −30

Generating CP is already a challenging task, and maintaining CP in a MIMO environment becomes even more difficult, especially when dealing with more than a single CP. Therefore, this research contributes to the field by addressing this novel aspect and providing insights into the impact of MC on CP generation in MIMO systems.

### 3.4 Comparison performance of 3 dB AR bandwidth between conventional and proposed DRA

**Fig. 16:**
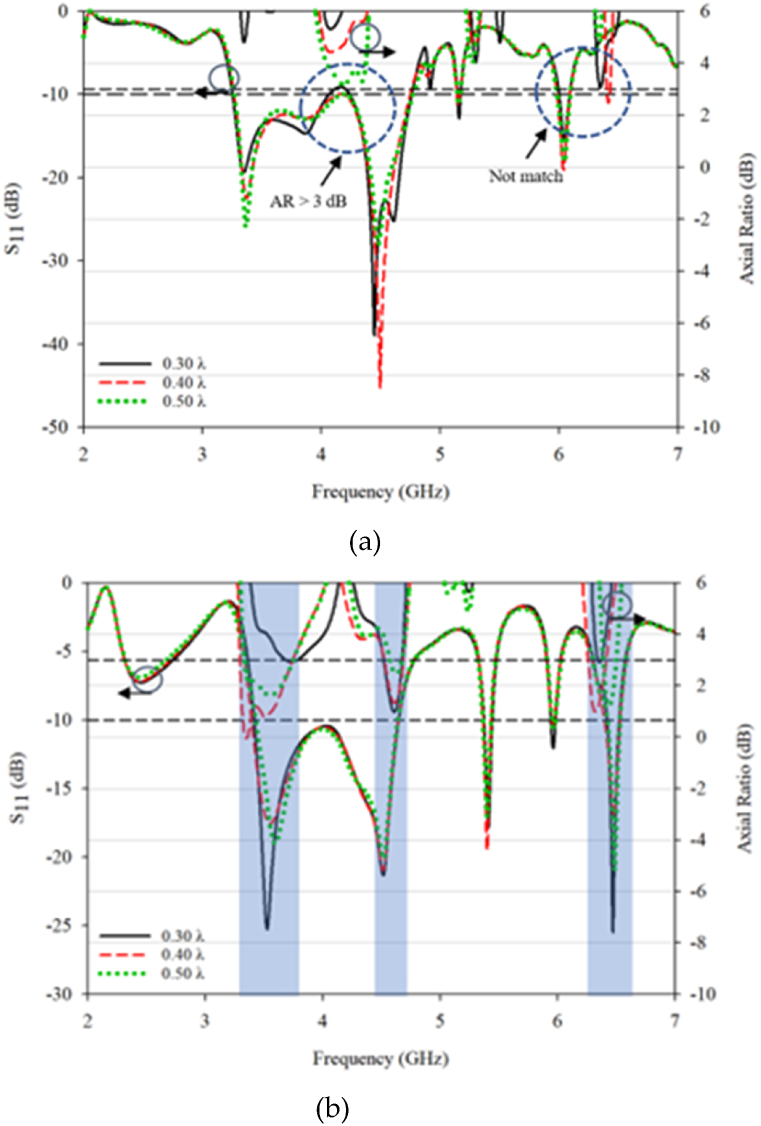
S_11_ and 3 dB axial ratio characteristic for MIMO: (a) conventional; (b) proposed DRA.

**Fig. 17:**
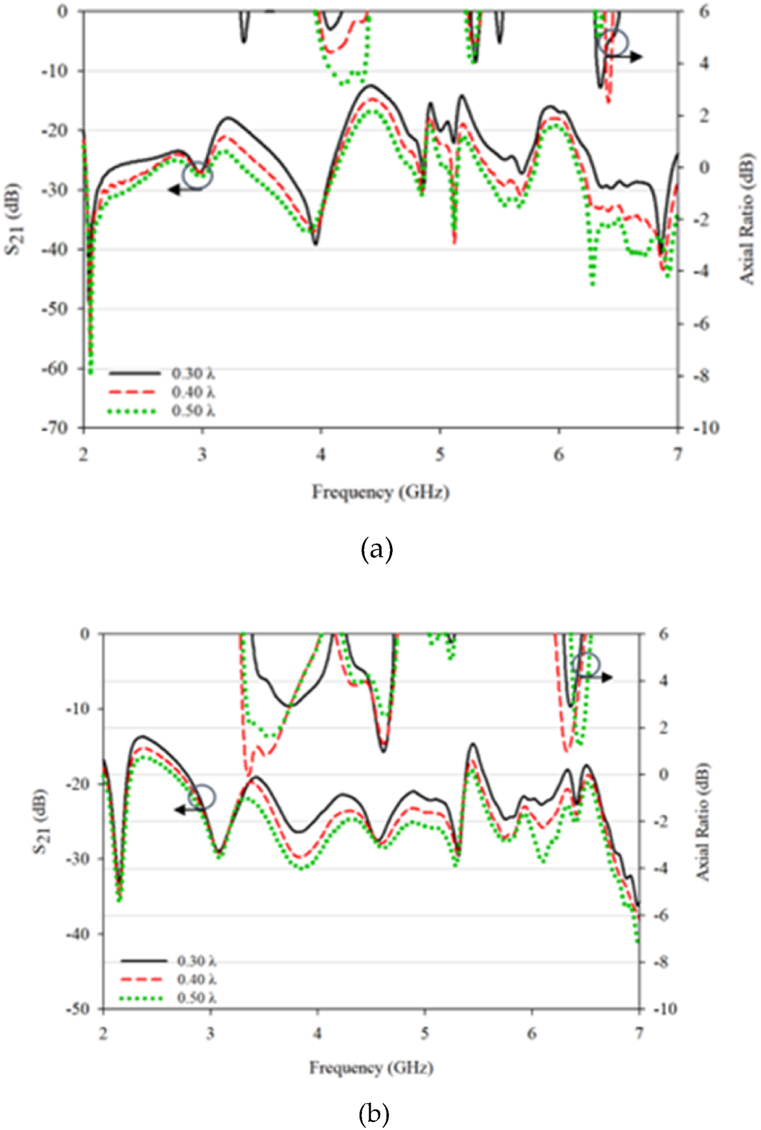
S_21_ and 3 dB axial ratio characteristic for MIMO: (a) conventional; (b) proposed DRA.

Simulated 3 dB AR bandwidth for the conventional and proposed DRA are presented in Fig. 16, Fig. 17. The peak value happens at a 0.30λ. The distance reduction affects the antenna performance, particularly the coupling between the DRA elements for both the conventional and proposed designs. However, better result can be seen with the proposed design, as it is able to produce triple band CP that well match to the operating frequencies with acceptable mutual coupling. The optimized performance was found at 0.30λ gap distance. In contrast, only one CP is produced in conventional RDRA at one resonant frequency of 4.2 GHz with a gap of 0.50λ between DRA elements and no CP is produced for other distances.

For the conventional design, the simulated S_11_ and S_21_ are in good agreement with producing results below −10 dB. When comparing the AR at different distances, the results are surprising as the AR is matched at one frequency, even though the impedance bandwidth is large. The proposed design produces a triple 3 dB AR bandwidth with a good value, which could potentially be used in 5G and WLAN coverage. The maximum distance for the proposed design is 0.40λ, and the proposed design is constructed and measured at 0.40λ for validation purposes.

**Fig. 18:**
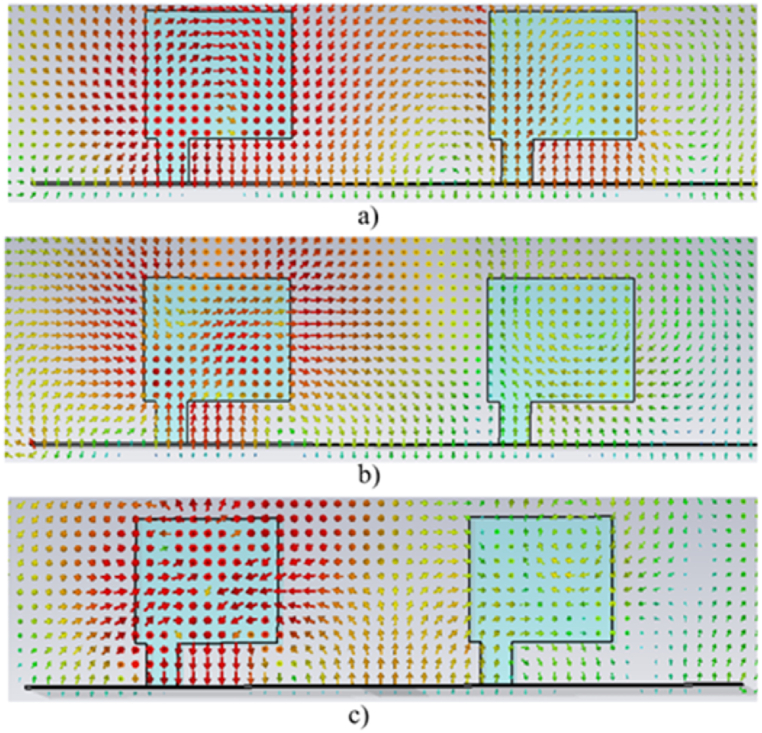
E- Field for the proposed design: (a) 3.3 GHz; (b) 4.6 GHz; (c) 6.3 GHz.

**Fig. 19:**
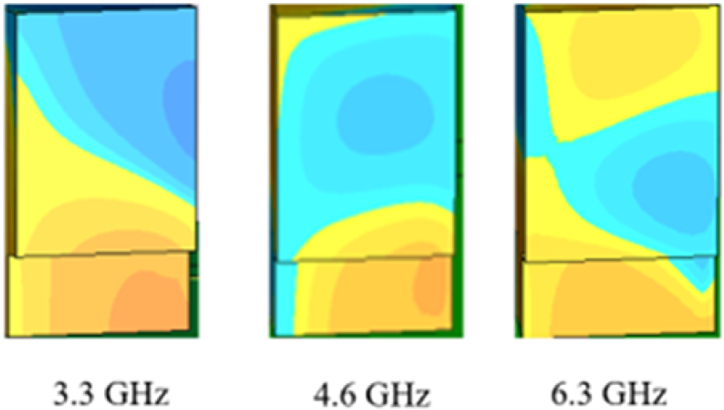
H-field for the proposed design.

Fig. 18, Fig. 19 illustrate E-field and H-field for the proposed design with the degenerate mode pair of the fundamental modes of TE_x_^δ21^, TE_x_^δ31^, and TE_x_^δ51^ at 3.3, 4.6, and 6.3 GHz.

**Fig. 20:**
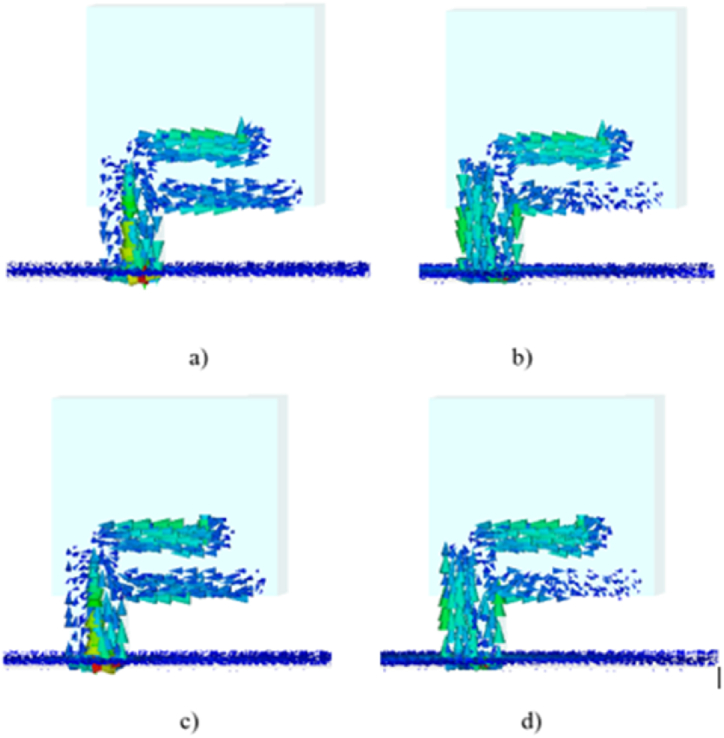
Surface current distribution at 3.57 GHz: (a) phi = 0°; (b) phi = 90°; (c) phi = 180°; (d) phi = 270°.

The computed vector surface current distribution is presented to further examine the CP. Fig. 20 shows the simulated surface current distribution on the metal feed and the parasitic patch for the anticipated antenna at a frequency of 3.3 GHz with varied phase angles of phi = 0°, 90°, 180°, and 270°. The figure displays that the vector surface current distribution was executed via rotation with various phase angles and rotations occurring in anti-clockwise orientation; reflecting the presence of left-handed CP (LHCP).

**Fig. 21:**
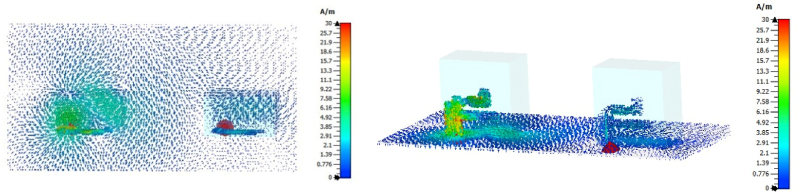
Surface current for conventional design.

**Fig. 22:**
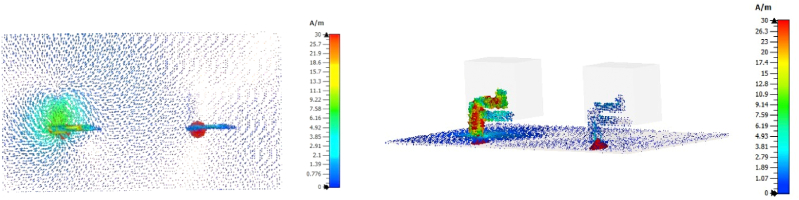
Surface current for the proposed design.

It is clear from Fig. 21, Fig. 22 that the conventional design has high surface current density on both DRs, while the proposed design, which uses trimming of the DRA to improve mutual coupling, significantly reduces the current density. The optimization from conventional DRA into proposed design improved the antenna performance particularly S_11_ and AR bandwidth.

## 4 Antenna simulation and measured result

The section focuses on two key aspects of the proposed prototype. (a) Experimental verification of the simulated effects, which involves testing and verifying the results of simulations in an anechoic chamber. The aim is to measure the reflection coefficient and radiation pattern of the prototype. (b) Investigation of diversity performances, which involves studying the performance of the prototype in terms of diversity, a technique used in wireless communication to improve signal quality and reliability. Fig. 25 provides a visual representation of the experimental setup used for the first aspect.

**Fig. 23:**
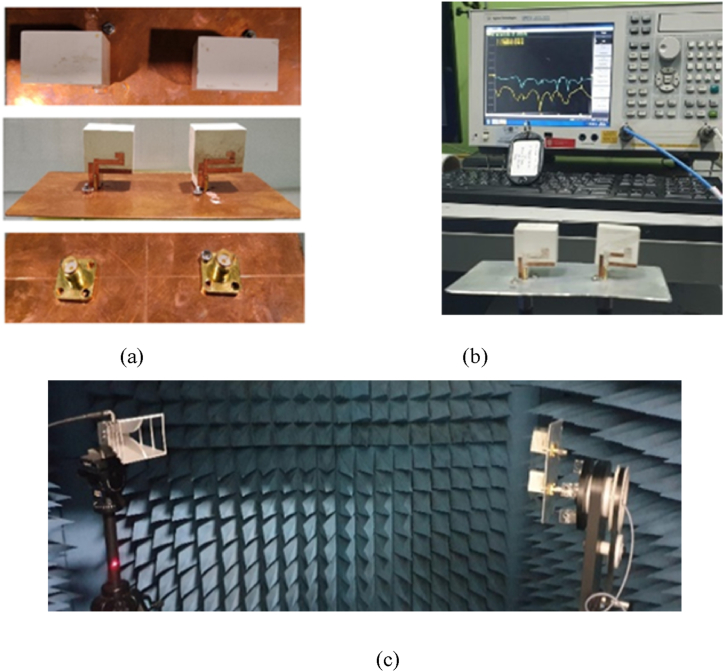
Fabricated MIMO antenna: (a) Front, top and rear view; (b) Antenna under test using VNA; (c) Anechoic chamber.

The development of antenna prototype for MIMO involves fabrication as in Fig. 23(a). To test the antenna's performance, a vector network analyzer is used to measure S_11_ and S_21_ parameters as in Fig. 23(b) while the radiation pattern and antenna radiation are measured in an anechoic chamber as in Fig. 23(c).

### 4.1 Comparison performance of S_11_, S_21_ and axial ratio (AR)

**Fig. 24:**
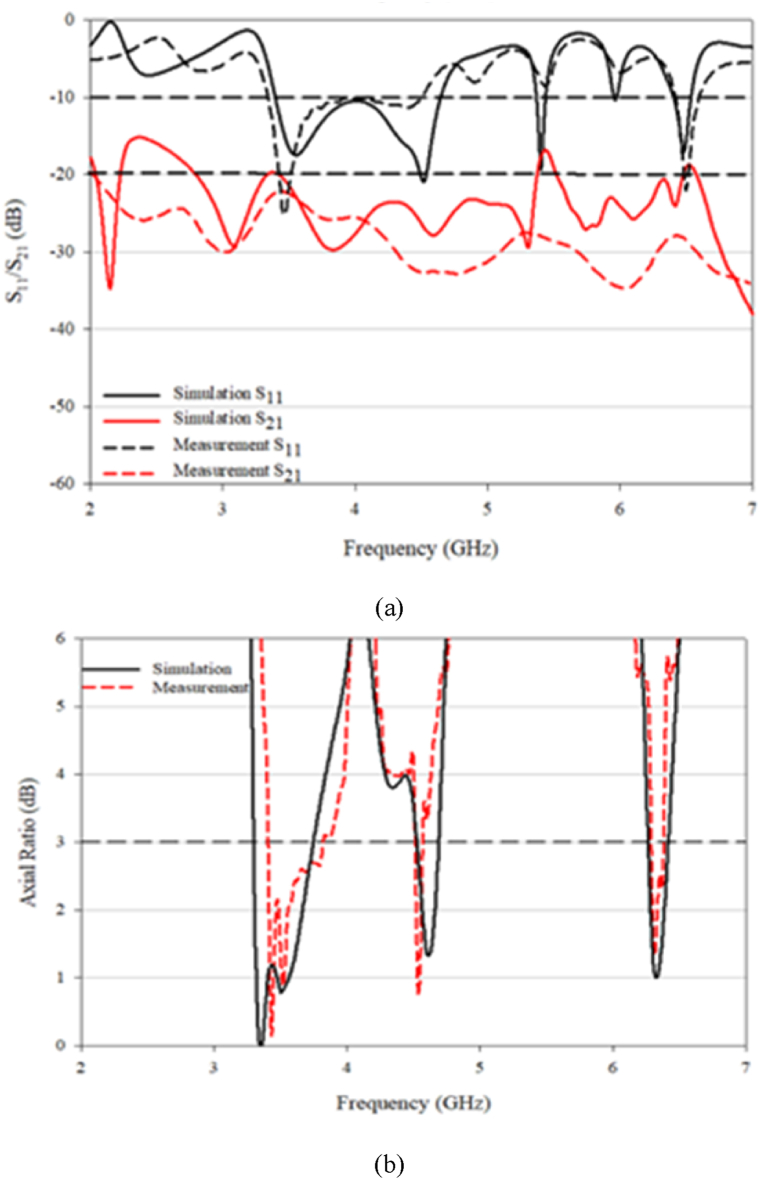
(a) S_11_ and S_21_ simulation versus measurement; (b) AR simulation versus measurement.

The results of the simulated and measured S_11_ and S_21_ for the proposed MIMO antenna are shown in Fig. 24(a). The comparison of the simulation and measurement results show close agreement. The antenna has an impedance bandwidth (−10 dB) of 13.88% (3.33–3.82 GHz), 2.96% (4.55–4.69 GHz), and 4.45% (6.26–6.54 GHz) and an AR bandwidth (3 dB) of 11.17% (3.41–3.81 GHz), 2.89% (4.56–4.69 GHz), and 2.48% (6.37–6.53 GHz). The isolation of the antenna over the entire operating frequency band is around −20 dB.

Fig. 24(b) shows the comparison of the measured and simulated AR of the proposed MIMO antenna. The simulation and measurement results are in close agreement, however, due to measurement accuracy limitations, there is a slight difference between the two. The main focus is to match the impedance bandwidth and AR bandwidth, and the results indicate that this has been achieved.

**Fig. 25:**
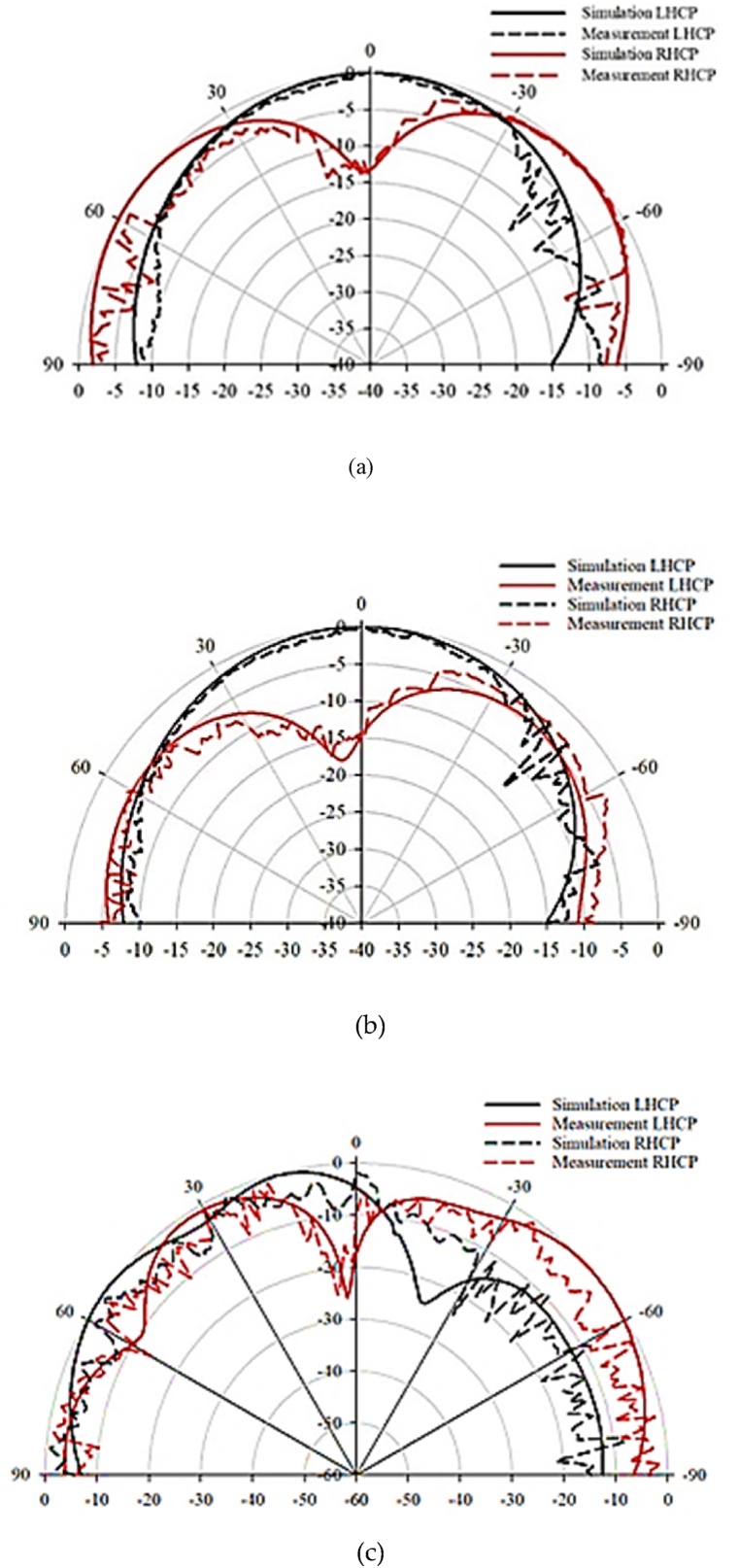
Radiation pattern at: (a) 3.5 GHz; (b) 4.5 GHz; (c) 6.3 GHz.

Fig. 25 compares the simulated and measured radiation patterns of the proposed DRA at frequencies of 3.6 GHz, 4.6 GHz, and 6.3 GHz. The antenna demonstrates left-hand circular polarization (LHCP) as the difference between LHCP and right-hand circular polarization (RHCP) patterns is at least −15 dB.

**Fig. 26:**
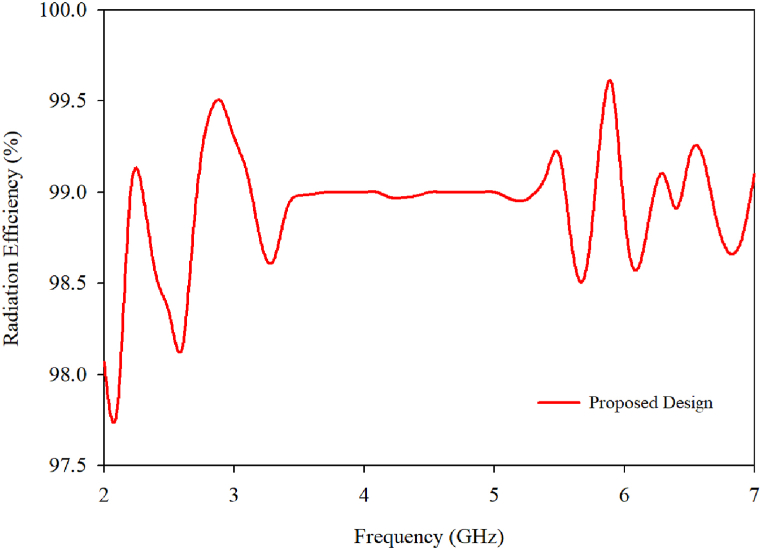
Radiation efficiency of the proposed MIMO antenna.

The antenna exhibits 90% radiation efficiency, indicating that the majority of the power at the antenna's input is being radiated as seen in Fig. 26.

### 4.2 Gain, radiation efficiency, mean effective gain (MEG), envelope correlation coefficient (ECC), diversity gain (DG), and total reflection coefficient (TARC)

**Fig. 27:**
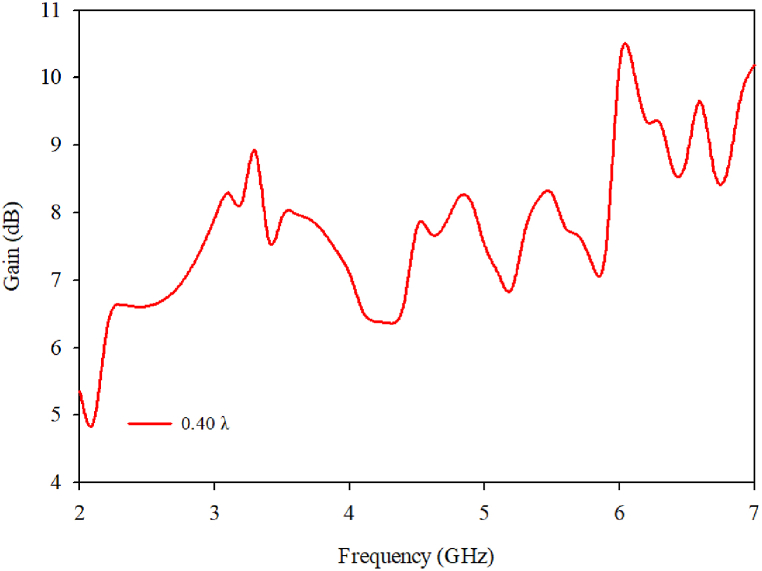
MIMO gain at 0.40λ.

Fig. 27 shows the gain of the proposed antenna as a function of the frequencies of ports 1 and 2. According to the results, the antenna's gain is seen to be greater than 3 dB for all resonance frequencies.

**Fig. 28:**
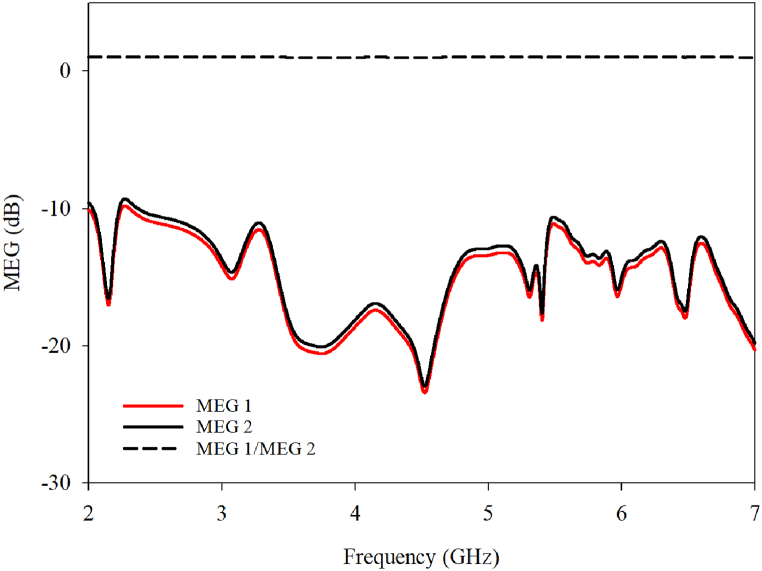
MEG for the proposed design.

MEG, or Multipath Environment Gain, serves as a crucial metric that assesses an antenna's ability to capture electromagnetic power in diverse environments. It is quantified as the ratio between the received power from the antenna's diversity antennas and that from isotropic antennas. This ratio, determined by equations 1 and 2 [16], illustrate the antenna's efficiency amidst multipath scenarios. For optimal functionality, the MEG across antenna ports should ideally remain below 3 dB. As depicted in Fig. 28, the proposed design operates within this threshold at its designated frequency, ensuring effective performance in real-world conditions

(1a) $${\text{MEG}}_{1}=0.5[1-{\left|S_{11}\right|}^{2}-{\left|S_{12}\right|}^{2}$$

(2a) $${\text{MEG}}_{2}=0.5[1-{\left|S_{12}\right|}^{2}-{\left|S_{22}\right|}^{2}$$

In order to examine further on MIMO performance, the evaluation of several diversity performance metrics has been highlighted in this section. The parameters employed to verify the effectiveness of the suggested MIMO antenna are the envelope correlation coefficient (ECC), diversity gain (DG), and total active reflection coefficient (TARC). The envelope correlation coefficient (ECC) measures the similarity between the envelopes of the signals received at two antennas, with a lower ECC indicating greater signal independence between the antennas and better diversity performance. Equation (3) is used to calculate the simulation and measured results of ECC [17].

(3) $${ECC}_{\rho_{e}}=\frac{\left|S_{11}^{*}S_{21}^{*}+S_{11}S_{22}\right|}{\left(1-{\left|S_{11}\right|}^{2}+{\left|S_{21}\right|}^{2}\right)\left(1-{\left|S_{22}\right|}^{2}+{\left|S_{12}\right|}^{2}\right)}$$

**Fig. 29:**
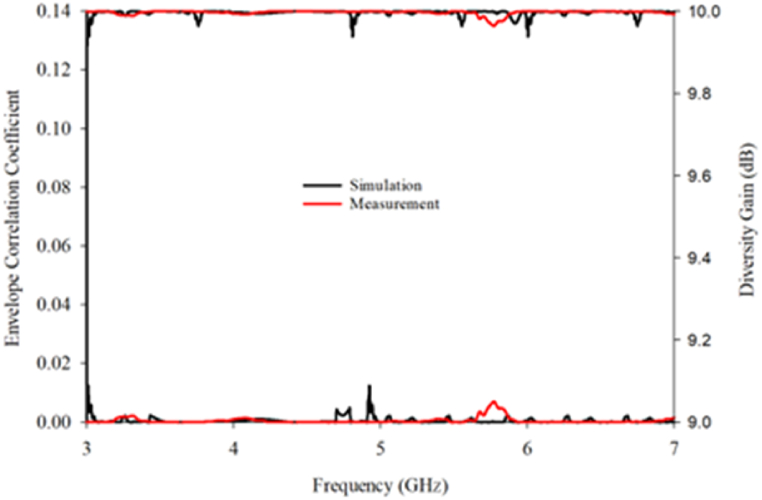
ECC and DG results.

For practical applications, an ECC limit of less than 0.5 is required. According to Fig. 29, the measured and simulated value of ECC at the overall frequency range is kept well below 0.2. Meanwhile, DG is another performance metric that measures the improvement in the signal-to-noise ratio (SNR) due to the use of multiple antennas. A higher DG value indicates better diversity performance. Equation (4) contains the formula for computing the DG [17].

(4) $$DG=10\;\sqrt{1-\left|ECC\right|}$$

The DG must be closer to 10 dB in ideal situations. According to Fig. 29, the suggested MIMO antenna's measured and simulated DG is over 10 dB at every required frequency range. It can be concluded that the suggested MIMO antenna, which has a smaller ECC and a larger DG, has good MIMO performance.

**Fig. 30:**
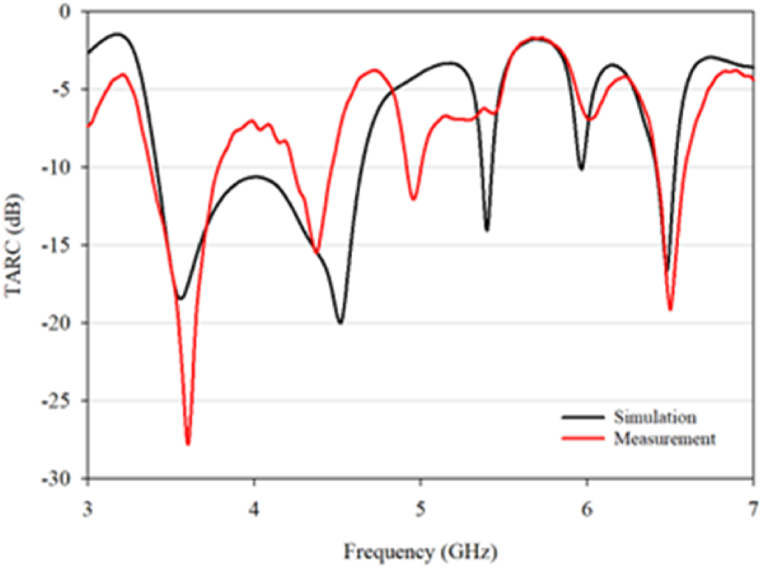
TARC.

Another evaluated parameter for the antenna is the TARC. TARC is a measure of the energy reflected back to the source in a multi-antenna system. A lower TARC value indicates better system performance, as less energy is being lost due to reflections as in Equation (5) [17]. For a two-port MIMO antenna, TARC is calculated using Equation (3) and must be below −0 dB. For the proposed MIMO antenna at three operating frequencies, the TARC is below −5 dB as seen in Fig. 30.

(5) $$\Gamma_{a}^{t}=\frac{\sqrt{\sum_{i}^{N}{\left|b_{i}\right|}^{2}}}{\sqrt{\sum_{i}^{N}{\left|a_{i}\right|}^{2}}}$$

Therefore, lower ECC, higher DG, and lower TARC values are desirable for better MIMO system performance and the proposed design meet these requirements. The evaluation of these metrics helps in understanding the effectiveness of the proposed MIMO antenna in a multi-antenna system.

**Fig. 31:**
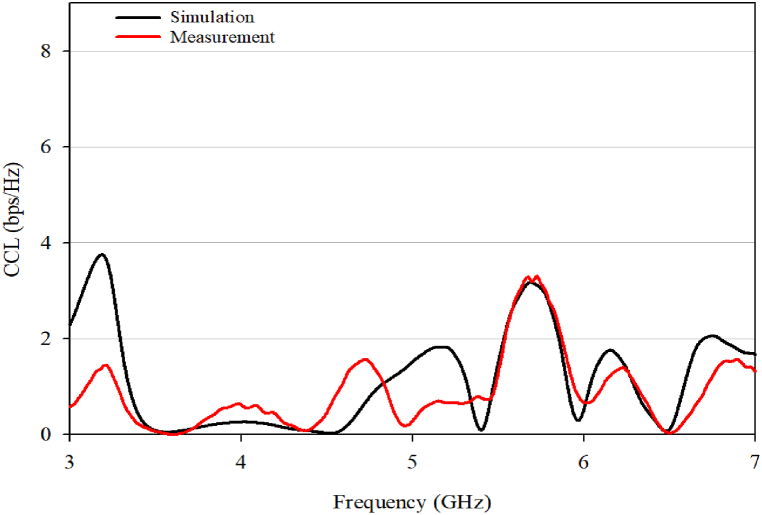
CCL.

Chanel Capacity Loss (CCL) result is shown in Fig. 31. In general, the channel capacity of the MIMO system varies linearly with an increasing number of used antenna elements. However, it also includes some losses due to the presence of correlation among the MIMO channels. The correlation among elements in MIMO channel systems produces a capacity loss. A low CCL value, less than 0.4bps/Hz, is desired for a MIMO system to ensure optimal performance.

## 5 Performance comparison between current MIMO antenna and other existing work

In summary, the proposed antenna in Table 5, Table 6 is compared with other related literature based on various criteria such as impedance bandwidth, overlapping CP BW, gain, size, and isolation for MIMO application. The results show that the proposed antenna has a unique advantage of exhibiting a triple band CP operation and simultaneous frequency and radiation pattern reconfiguration capabilities in the same structure, which is not reported in the existing literature [9,[18], [19], [20], [21], [22]]. The author mentions in Ref. [18] that only the impedance bandwidth is considered, but the overlapping bandwidth is not discussed. In contrast [23], mentions that the impedance band produces three bandwidths but the overlapping bandwidth is only one. It is commonly known that a triple band CP is obtained from a single element, as seen in Refs. [[16], [24], [25], [26]]. In conclusion, when comparing the overlapping CP bandwidth, the proposed antenna shows improved results as compared to a single element. This indicates that the proposed antenna is capable of adapting to changes in distance while maintaining optimal performance for MIMO applications.

**Table 5:** Comparison from previous research for single element.

Ref.	Range (GHz)	Impedance BW (%)	Overlapping CP BW (%)	Gain (dBic)
15	1.92–1.955	1.55	1.55 (1.92–1.955)	1.2
16	2.315–2.5	7.68	4.96 (2.36–2.48)	1.6
	3.415–3.55	3.87	0.8 (3.502–3.53)	−1.5
	2.43–4.35	48	3 (3.18–3.29)	6.2
	4.82–5.61	14.8	4.5 (4.88–5.18)	9.44
	–	–	3.18 (5.28–5.18)	9.3
17	2.12–2.45	14.4	6.34 (2.29–2.44)	4.48
	2.64–3.98	40.5	6.65 (2.76–2.95)	5.38
	–	–	7.09 (3.67–3.94)	5.75
18	2.06–2.707	25.28	11 (2.29–2.61)	6.2
	4.847–5.78	22.61	0.61 (4.847–4.94)	9.44
	8.06–8.7	7.6	0.97 (8.23–8.31)	9.3
This paper	3.3–4.69	35.4	11.27 (3.3–3.68)	6.8
	5.36–5.44	1.74	12.18 (4.17–4.69)	7.6
	6.41–6.55	1.85	3.52 (6.44–6.55)	8.5

**Table 6:** Comparison from previous research for MIMO.

Ref.	Range (GHz)	Impedance BW (%)	Overlapping CP BW (%)	Gain (dBic)	Isolation (dB)
20	1.63–1.84	12.1	–	5.5	15
	2.43–2.71	10.89	–	5.9	–
	3.28–3.73	12.84	–	6.9	–
21	3.1–3.75	19	5 (3.43–3.6)	6.8	16.5
	5.3–5.6	9.4	2 (5.45–5.55)	4.6	16.2
22	2.38–2.52	–	5.7	–	11
23	2.21–3.13	34.45	4.18 (5.62–5.82)	1.5	20
	3.4–3.92	14.2	–	4.1	–
	5.3–6.1	14	–	2.3	–
24	5.15–6.12	–	5.30–5.87	–	17
9	3.50–4.95	38.51	20.82 (3.58–4.40)	6.5	26
This paper	3.41–4.65	34	11.66 (3.3–3.82)	8.3	∼20
	5.36–5.44	1.82	3.04 (4.55–4.69)	7.8	–
	6.25–6.53	1.95	2.22 (6.25–6.53)	9.9	–

Overall, the proposed compact antenna has a low ECC value and a high channel capacity, which contributes to its overall efficiency. The comparison study shows that obtaining all of these features concurrently for MIMO applications is challenging in previous MIMO antenna designs due to size limitations and complexity. However, the proposed antenna successfully achieves the desired MIMO parameters and has the added advantage of being multiband with a simple and practical design.

## 6 Conclusion

In summary, a MIMO DRA with triple band characteristics has been designed, fabricated, and tested. The parasitic patch and metal strip is used to achieve the desired frequency bands of 3.3, 4.6, and 6.3 GHz. The DRA structure was slightly trimmed to obtain a wider band at three different frequencies while maintaining good matching at the operating frequency with reduced in mutual coupling. The results show that the proposed antenna has better antenna gain, good impedance bandwidth, polarization diversity, and a low ECC value of less than 0.02%, demonstrating its good performance for MIMO application.

## Data availability

Data will be made available on request.

## CRediT authorship contribution statement

**A. Ali:** Writing – original draft, Investigation, Formal analysis. **M.N.M. Yasin:** Validation, Supervision, Resources, Conceptualization. **I. Adam:** Writing – review & editing, Supervision, Methodology, Investigation. **A.M. Ismail:** Visualization, Software, Data curation. **S.P. Jack:** Writing – review & editing, Validation. **Abdullah Alghaihab:** Resources, Funding acquisition. **N.I.M. Nor:** Writing – review & editing, Validation. **N.A.A. Rahman:** Writing – review & editing, Validation.

## Declaration of competing interest

The authors declare that they have no known competing financial interests or personal relationships that could have appeared to influence the work reported in this paper.
